# The changes in female physical and childbearing characteristics in china and potential association with risk of breast cancer

**DOI:** 10.1186/1471-2458-12-368

**Published:** 2012-05-21

**Authors:** Qiang Zhang, Li-yuan Liu, Fei Wang, Kun Mu, Zhi-gang Yu

**Affiliations:** 1 Department of Breast Diseases, The Second Hospital of Shandong University, Jinan, Shandong Province, China; 2 Epidemiology Institute, School of Public Health, Shandong University, Jinan, Shandong Province, China; 3 Department of Pathology, School of Medicine, Shandong University, Jinan, Shandong Province, China

## Abstract

**Background:**

There has been a sharp increase in the incidence of breast cancer in China in recent years. A number of female physical characteristics, such as age at menarche, menopause, first birth and the duration of breastfeeding, have been linked to breast cancer, yet data on these factors in Chinese women is largely missing both for aggregate and age-specific data. Thus, the objective of this study was to explore changes in female menstrual and childbearing characteristics as a possible explanation for increasing rates of breast cancer in this country.

**Method:**

From July to September 2008, a population based cross-sectional breast cancer survey covering 124,758 females from 4 provinces or cities in Eastern China was carried out, using multi-stage and cluster methods. In-person interviews based on a self-designed structured questionnaire were performed, in which female physiological and reproductive factors, such as age at menarche and menopause, menstrual cycle history, childbearing history, breastfeeding methods, abortions or miscarriage, were included. For every 10-year age category, the subjects were divided, and across those age groups, all the above factors were compared respectively and changes in physical and childbearing characteristics were evaluated. ANOVA was used to compare the differences across the groups.

**Results:**

A total of 122058 subjects were included in the final analysis. The mean age at menarche was 15.39 years, the mean number of full-term pregnancies was 1.58, the mean duration of breastfeeding was 22.68 months, the mean age at first birth was 23.75 years, the mean frequency of miscarriage was 0.36, and the mean age at menopause was 48.63 years. Significant differences across the several age groups were noted for the age at menarche, number of full-term pregnancies, accumulated duration of breastfeeding, age at first birth, number of miscarriages, and age at menopause. These data clearly showed a gradual shift towards an earlier age at menarche, fewer pregnancies and shorter breastfeeding lengths.

**Conclusions:**

Significant changes in female physical and childbearing characteristics across a number of different age ranges were detected. These changes may be related to the increasing trend of breast cancer in China.

## Background

Breast cancer is one of the most prevalent malignancies in women worldwide. In 2008, 1.38 million people worldwide, mostly from Europe and the USA, were diagnosed with breast cancer [1]. Compared to Western countries, the incidence of breast cancer in China has historically been low, although in recent years there has been a sharply increase trend in the incidence of breast cancer in China. For instance, work by Yang et al., showed a 38.5% growth in the number of breast cancer cases between 2000 and 2005 and a 37.1% increase in mortality due to breast cancer in that same period [2]. In this context, a substantial body of international and domestic research has focused on the factors associated with breast cancer. However, these studies have largely been based on clinical cases and have had other limitations such as small sample sizes, different ethnic groups within the target population and differences in regional social-economic conditions and customs, which resulted in inconsistent conclusions [3-7]. Traditionally, female physical characteristics, such as age at menarche, menopause, when an individual gives birth and their breastfeeding period, have been thought of as being linked to breast cancer [8]. Yet to date, data on these physical characteristics in Chinese women has been absent, both with respect to total values and for age-specific groups. In this study, to better understand the incidence rate, characteristics and risk factors for breast cancer in Chinese women, a cross-sectional survey covering 122,058 females in 4 provinces or metropolitan areas was performed in Eastern China between July 15 and September 15, 2008. In this study, data on several physical characteristics including those related to menstrual, marital and childbearing characteristics were also evaluated and changes in these characteristics during the past years were also. Therefore, in this paper we present data regarding changes in female menstrual, marital and childbearing characteristics in women from Eastern China.

## Methods

### Study populations

**Figure 1:**
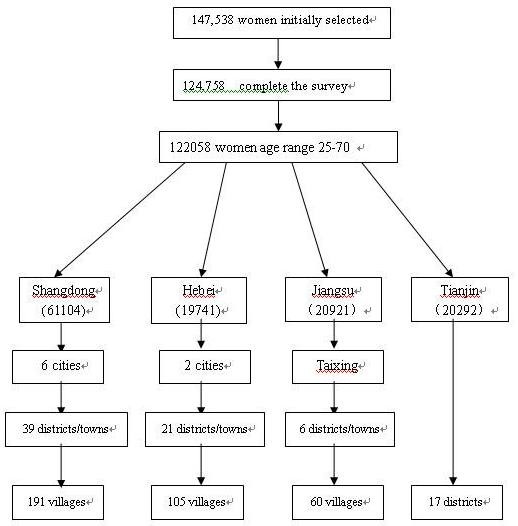
**Schematic diagram illustrating the overall study design**. Random samples were obtained through multi-stage stratified cluster sampling. Provinces, counties or regions, villages or communities were successively randomly selected.

Random samples were obtained through multi-stage stratified cluster sampling. The target population included 25 to 70-year-old females of the Han ethnic group who at the time of survey had been registered for over two years at a local residence and had lived at least six months in that local residence. Long-term migrant workers were excluded. The provinces in Eastern China where the Han ethnic group mainly resides, include Shandong, Jiangsu, Hebei and Tianjin, were selected as the survey provinces. Subsequently, counties or regions, villages or communities were successively randomly selected. Women who met the study requirements were selected for the survey. A schematic outlining the sampling procedure is shown in Figure 1.

### Study design

This study was a cross-sectional epidemiological survey, including face-to face interviews and breast examinations. Data were collected through in-person interviews based on a self-designed structured questionnaire, in which female physiological and reproductive factors, such as age at menarche, age at menopause, menstrual cycle history, childbearing history, breastfeeding methods, history of abortions or miscarriage, contraceptive methods and the use of contraceptive medications were included. For all of the variables covered by the questionnaire, the answers were defined by strict criteria. For example, menopausal status was divided into premenopausal and postmenopausal refer to the criteria the National Comprehensive Cancer Network (NCCN) in 2008 [9]. If the subject had experienced continuous amenorrhea for over 12 months without physiological(gestation or breast-feeding) and pathological reasons(oral contraceptive or hormone therapy) or the subject was over 60 years or the subject had experienced oophrectomy, she was defined as being menopausal, but we didn’t examine the serum FSH and estradiol. Finally, all data was stratified according to age (25-34, 35-44, 45-54, 55-64, 65-70 years) for the age specific analysis.

### Implementation

As a cross-sectional study, all interviews were completed in 2 months: from July 15 to Sept 15, 2008. And before the survey began, this study was approved by the ethics committees of the second hospital of Shandong University and those at the collaborating institutions in each region (all the official seals and the signature of committee members are available, but there is no ethics reference number) and informed consent was obtained from all of the study subjects. Surveyors and physicians were recruited in each survey region before the implementation of this study. A total of 77 physicians and 248 surveyors participated in the on-site survey process.

All study subjects were strictly selected according to random sampling methods, and the final sampling concordance rate was 97.8%. During the survey process, all interviews were under strict quality control. Firstly, a supervisor in each region oversaw the surveyors and sampled their work for inspection. Further, 10% of the questionnaires completed at the end of each day were randomly sampled to inspect for their completeness, accuracy and standardization. After the quality assessment was completed, all identified errors and missed survey items were promptly corrected. If important information was found to be missing in the questionnaire, the interviewer would collect the missing information from the original survey site the next day. Finally, after the completion of the survey, sampled re-screening was performed for each survey site, and the results showed a consistency of 92.19%.

### Statistical analysis

Epidata 3.1 (EpiData Association, Odense, Denmark) was used to establish the database. Analysis of variance was used to compare differences across different age groups with different variables. The liner regression analysis was used to performe the trend test in age groups and Age at menarche, Number of full-term gestations, Accumulative breastfeeding periods . SPSS 16.0 (SPSS Inc., Chicago, IL, USA) was used to carry out the data analysis. Tests of significance were evaluated against the null hypothesis of no association, using P < 0.01 as a cut-off for significance.

## Results

### Overall population characteristics

In this survey, 147,538 women were initially selected, and of these women, 124,758 were approached to undertake the survey, giving a 15.44% rate of loss. A total of 124,758 women from Shandong, Hebei, Jiangsu province and the Tianjin metropolitan area were registered and interviewed. Of these 1037 subjects were younger than 25 years, 1629 subjects were older than 70 years. A further 34 were excluded due to a loss of important information. Thus, the final total study population was 122,058 (sampling concordance rate: 97.84%). Among these women, 61,104 women were come from Shandong province, amounting to the 0.64% of Shandong province’s total population and 1.29% of Shandong province’s total female population; 19,741 women were from Hebei province, amounting to 0.27% of Hebei’s total population and 0.56% of it’s female population; 20,921 women were from Jiangsu province, representing 0.27% of Jiangsu province’s total population and 0.5% of its female population; 20,292 women were from the Tianjin metropolitan, amounting to1.56% of Tianjin’s total population and 3.33% of its female population [10].

The mean age for all subjects was 44.2 years (standard deviation, SD = 11.6). Of the total cohort, 85,755 (70.26%) of the subjects were premenopausal, while 36,303 (29.74%) were postmenopausal, with a mean age of 48.76 years (standard deviation, SD = 4.11) at menopause. The mean age at menarche was 15.39 years (SD = 2.02) for all subjects. Overall, 118,623 subjects had given birth at least once, and 95,434 (78.6% of 118 623) had breastfed for at least 12 months. There were 88,632 subjects from rural areas and 33,696 from urban areas, giving an urban/rural proportion of 0.38, which is similar to that found for China as a whole. Characteristics of the population, both in total and for each age group, are shown in Table 1.

**Table 1:** Population characteristics, including the proportion of subjects recruited from each region in Eastern China, the location, age at menarche, the frequency of full-term births and miscarriages, and the proportion of pre- and post-menopausal women

***Characteristics***	***Participants***	**Proportion (%)**
**Regions**		
Shandong	61104	50.06
Jiangsu	20921	17.14
Hebei	19741	16.17
Tianjin	20292	16.62
Loss value	0	
**Location**		
Urban	33696	27.61
Rural	88362	72.39
Loss value	0	
**Age of menarche**		
7-11	867	7.10
12-13	19395	15.89
14 or more	101744	83.36
Loss value	52	
**Frequency full-term gestation**		
<2	70719	57.94
> = 2	51339	42.06
Loss value	0	
**Number of miscarriages**		
0	91433	74.91
1	20632	16.90
2	7398	6.06
>2	2427	1.99
Loss value	168	
**Menopausal status**		
Pre-menopause	85755	70.26
Post-menopause	36303	29.74
Loss value	0	

### Age at menarche

After excluding 162 women due to a loss of age data and 52 women due to a loss of menarche data, 121,844 subjects remained in the study. As shown in Table 2, the mean age at menarche for the 121,844 subjects included for analysis was 15.39 years. There were statistically significant differences across several of the age groups (*p* < 0.001). Trend test showed the age at menarche bring forward 0.479 years every 10 years young (*p* < 0.001), clearly indicating a gradual change towards an earlier age at menarche.

**Table 2:** Changes in the mean age at menarche, the mean length of full-term gestation and accumulated duration of breastfeeding in women from Eastern China

**age group**	**age at menarche(year)**			**Times of full-term gestations**			**Accumulative breastfeeding period (months)**		
	**number**	**mean ± SD**	P value*	**number**	**mean ± SD**	P value*	**number**	**mean ± SD**	P value*
25-34	27217	14.65 ± 1.57		27226	1.01 ± 0.48		26749	13.62 ± 9.80	
35-44	40812	15.12 ± 1.76		40832	1.35 ± 0.57		39823	18.99 ± 13.85	
45-54	27079	15.68 ± 2.12	<0.001	27092	1.67 ± 0.78	<0.001	26518	24.86 ± 21.35	<0.001
55-64	19631	16.26 ± 2.30		19641	2.20 ± 1.00		19252	32.39 ± 28.62	
65-70	7105	16.20 ± 2.36		7105	3.06 ± 1.53		6953	43.51 ± 39.50	
total	121844	15.39 ± 2.02		121896	1.58 ± 0.94		119295	22.68 ± 21.78	
loss value	214			162			2763		

*Statistical analysis was performed using ANOVA

### Number of full-term gestations

After excluding 162 women loss of age data, 121,896 women were left to be analyzed. As shown in Table 2, the mean frequency of full-term gestations for each of the 121,896 subjects was 1.62. Moreover, statistically significant differences across the several age groups (*p* < 0.001) were observed, trend test showed the number of full-term gestations decrease 0.441 every 10 years young(*p* < 0.001),clearly indicating a gradual shift to fewer gestations.

### Accumulative breastfeeding periods

After excluding 162 women loss of age data and 2,601 women due to a loss of breastfeeding data, 119,295 women remained. As shown in Table 2, for the 119,295 subjects the average duration of breastfeeding was 22.68 months. Significant differences across a number of the age groups (*p* < 0.001) were noted, trend test showed the accumulative breastfeeding periods decrease 4.50 months every 10 years young(*p* < 0.001), indicating gradual shift to shorter breastfeeding periods.

### Age at first birth

Of the 121,896 women to be analyzed, as shown in Table 3, the mean age at first birth was 23.75 years. Significant differences across a number of the age groups (*p* < 0.001) was also noted.

**Table 3:** Changes in mean age at first birth, the frequency of miscarriage and mean age at menopause in women from Eastern China

**age group**	**Age at first birth (years)**			**Times of miscarriage**			**Age at menopause**		
	**number**	**mean ± SD**	P value*	**number**	**mean ± SD**	P value*	**number**	**mean ± SD**	P value*
25-34	27226	22.38 ± 7.73		27164	0.29 ± 0.65				
35-44	40832	24.36 ± 3.54		40777	0.42 ± 0.77				
45-54	27092	24.39 ± 2.94	<0.001	27065	0.45 ± 0.83	<0.001	9955	47.92 ± 4.60	
55-64	19641	23.95 ± 3.50		19626	0.27 ± 0.65		18118	49.28 ± 4.29	<0.001
65-70	7105	23.04 ± 4.00		7098	0.22 ± 0.61		6724	48.71 ± 4.62	
total	121896	23.75 ± 4.80		121730	0.36 ± 0.74		34797	48.63 ± 4.82	
loss value	162			328			1506		

*Statistical analysis was performed using ANOVA

### Number of miscarriage

After excluding 162 women due to a loss of age data and 168 women due to a loss of miscarriage data, there were 121,730 women left to be analyzed (2 women were excluded due to a loss of both age and miscarriage data). As shown in Table 3, the subjects suffered an average of 0.36 miscarriages, and a significant between a number of the age groups (*p* < 0.001) was also observed.

### Age at menopause

A small number of women in the 25-34 and 35-44 age groups had gone through menopausal as a result of medical conditions or procedures and were excluded from the final analysis, leaving a total population of 34,797. As shown in Table 3, the mean age at menopause for the subjects was 48.63 years. a number of significant differences across the different age groups (*p* < 0.001) were detected.

## Discussion

In 2008, 1.38 million women across the world were diagnosed with breast cancer, resulting in 458 000 deaths. Of this number, 170,000 cases were in China, resulting in 44,000 deaths. Globally, 23% of cancer in women is due to breast cancer, making it the most common type of cancer in women. Data published by the WHO shows that the standardized incidence (per 100,000 women) of Chinese female breast cancer in 2008 was 21.6, which is much higher the figure of 16.4 observed in 2000. This data indicates that there has been a continuous increase in the incidence of breast cancer in China [1]. Based on the above information, we undertook a cross-sectional investigation in order to understand whether there have been changes in several of the known risk factors for breast cancer. The study featured women from Shandong, Jiangsu, Hebei, and Tianjin, as this north-eastern region of China is characterised by people of the Han ethnic origin. Our investigation included representative parts of the Chang-Jiang Area and the Yellow-River Area, including littoral, upland, plain, and metropolitan areas. Multi-stage stratified and cluster sampling methods were used to obtain the sample population from these four areas. To the best of our knowledge, this is the first large-scale, multiple-province epidemiological investigation of the risk factors for breast cancer in China since the 1970s.

The mean age at menarche in this study was 15.39 years. A number of statistically significant differences across the several age groups were observed, indicating a gradual shift in the age of menarche, towards an earlier age. In their study, Tianlin et al., studied age of menarche in a cohort of female Chinese students from 1985-2000, and found that approximately every 10 years the age of menarche was brought forward by about 2.5 months for urban women and 4.6 months for rural women. In their assessment of their findings, the shift to an earlier age at menarche was mainly attributed to economic development and increasing incomes [11]. However, in other studies, an early age at menarche is thought to be related to breast cancer. In a study by Gail et al.aimed at American females, it was found that that white women with the age at menarche before 12 carried an increased relative risk (20%) of breast cancer than those who underwent menarche after 14 [12], with an even greater increase in relative risk (30%) in black women [13]. A meta-analysis by Junqing Zhang et al., also showed that a later age of menarche was a protective factor of breast cancer and that the incidence of breast cancer in women who underwent menarche after 13 was 0.54 times that of those who underwent menarche before the age of 13 [14]. In our study we observed a gradual tendency towards a younger age at first menarche, suggesting that Chinese women may be gradually increasing their risk of breast cancer. These findings are concordant with the increasing breast cancer incidence observed in China over the last several years.

Our study also showed that fewer Chinese women were reaching full-term gestation, as revealed by our finding that more women in the 65-70 age reached full-time gestation than those in the 25-34 age group by an average of 1.94. This finding maybe related to the one child policy of China. The relationship between gestation and breast cancer is controversial, with most studies supporting the view that increasing birth number has a positive effects on breast cancer. Some studies have also shown that every pregnancy could reduce the risk of breast cancer by 7% [15]. A study by Tavani et al. study focused on women below 40 and showed that women with fewer gestations(≤3) bore higher risk of breast cancer than those without gestations or more gestations( ≥ 4) respectively [16]. In contrast, work by Junqing Zhang et al., revealed no relationships between the number of pregnancies and breast cancer [14].

In this study we also showed that accumulative breastfeeding periods were shorter in the younger age group, and this decreasing trend was linked to changes in full-term pregnancy, suggesting that the decreased length of breastfeeding periods was because women were, on average, having fewer pregnancies. Most studies support the view that breastfeeding is a protective factors for breast cancer. A study covering 150,000 females in 30 countries conducted by the Breast Cancer Estrogen Intergroup showed that for breastfeeding (in excess of 12 months) could reduce the risk of breast cancer by 4.3%, and that breastfeeding is an independent risk factor for breast cancer, and is not affected by other confounders such as age at first birth, parturition times, or race [17]. Studies from China have made similar findings [14,18]. Therefore, based on our findings and on the available literature, it is possible that the decreasing duration of breastfeeding due to reduced pregnancies may partly contribute to the increasing incidence of breast cancer in China.

Our study showed that the mean age at first birth was 23.75 years old. Of this, women from the 34-54 age group were the oldest at the time of first birth, followed by those from the 55-age group and then the 25-34 age group. This finding may be a reflection of policy changes. The Chinese government began implementing laws related to family planning, which advocated for late marriage and late childbirth, in late 1970s. Under this policy, all married women, were not be permitted to have a child without a baby birth permit. Thus, women aged between 34 and 54 may have delayed first birth. However, in the last 10 years, with the baby birth permit system nearly repealed and the easing of Family Planning Policy, women below 34 began to have their first birth at a much earlier age. Given that many studies have shown that an earlier age at first birth may reduce the risk of breast cancer, this demographic change has important implications. For instance, research has shown that white American women are at a 25% increased risk of breast cancer for every 4 years of delayed first pregnancy [15], but that there is no such pattern for African-American women [13]. Therefore, in their model, age at first birth was adopted as an independent factor but the model was adapted for each of the different races examined. However, there is no clear understanding on how this relates to China. Although some authors support the protective effect of earlier first birth [14], others have found no relationship [18].

This study showed that Chinese women suffered an average of 0.36 miscarriages, of whom women from the 35-54 age group experienced the most miscarriages. Given that numerous studies have shown that neither spontaneous nor artificial abortion is related to breast cancer [19], it is possible that this finding is also related to government policy. However, work by both Zhang and Zeng has shown that miscarriage is a breast cancer risk factor [14,18].

With respect to menopause, our study showed in Chinese women, the mean age at menopause was 48.63 years, and there were statistically significant differences across different age groups, among which women from 55-64 age group underwent menopause the latest. It is generally understood that delaying menopause increases the risk of breast cancer suggesting that women who underwent menopause below the age of 45 would have a decreased relative risk (by about 30-50%) than those who underwent breast cancer after 50 [20]. Work by Zhang also revealed age at menopause to be a risk factor for breast cancer [14].

## Conclusions

The findings of this study clearly showed a gradual shift to an earlier age at menarche, decreased duration of pregnancy and shorter breastfeeding periods. On the basis that the above determinants are traditional risk factors of breast cancer, we therefore concluded that these shifts in female physiology and reproductive characteristics are probably linked to the increasing incidence of breast cancer in China. This study was the first large-scale epidemiological investigation in China in the last 30 years, and such findings are important for further research on the treatment and prevention of breast cancer in this country, and contribute significantly to public health.

## Competing interests

The authors declare that they have no competing interests, including financial and non-financial interests.

## Authors’ contributions

QZ participated in the study design, population survey, data interpretation and draft the manuscript. LYL participated in the study design, population survey and statistical analysis. FW participated in the population survey, data collection and helped to draft the manuscript. KM participated in the population survey and data collection. ZGYwas the chief designer and executor of the programme and contributed the most to the data interpretation. ZGY had full access to all of the data in the study and takes responsibility for the integrity of the data and the accuracy of the data analysis. All authors read and approved the final manuscript.

## Pre-publication history

The pre-publication history for this paper can be accessed here:

http://www.biomedcentral.com/1471-2458/12/368/prepub
